# Energy efficiency enhancement in two European data centers through CFD modeling

**DOI:** 10.1038/s41598-025-11048-0

**Published:** 2025-07-10

**Authors:** Onur Muhammed Sarikaya, Mustafa Kuzay, Ender Demirel, Simon Pommerencke Melgaard, Jesper Ellerbaek Nielsen, Rasmus Lund Jensen, Ariel Oleksiak, Wojciech Szeliga

**Affiliations:** 1 Design and Simulation Tech. Inc, 26480 Eskisehir, Turkey; 2 Department of Civil Engineering, Eskisehir Osmangazi University, 26480 Eskisehir, Turkey; 3 Department of The Built Environment, Aalborg University, Thomas Manns Vej 23, Aalborg East, Denmark; 4 Poznan Supercomputing and Networking Center, Al. Jana Pawla II 10, Poznan, Poland; 5 Faculty of Mechanical Engineering, Poznan University of Technology, 3 Piotrowo street, Poznan, Poland

**Keywords:** Data center, KPI, Efficiency assessment, CFD, Energy efficiency directive, Mechanical engineering, Civil engineering

## Abstract

The new reporting scheme adopted by the European Union focuses on the efficiency assessment of data centers based on key performance indicators (KPIs). This has the potential to lead to further measures for improving energy efficiency of data centers. Consequently, a systematic approach is crucial for assessing and improving data center performance. This study investigates efficiency assessment of two European pilot data centers, located in Denmark and Poland, according to the KPIs calculated through Computational Fluid Dynamics (CFD) simulations of airflow and thermal structures. Two separate experimental approaches were adopted at the pilot sites. One measured airflow and temperatures using external sensors at server inlets and outlets, while the other measured inlet temperatures from embedded server sensors. The good agreement observed between simulated and experimental data confirmed the accuracy of the computational model. The validated CFD model was applied to investigate efficiency improvement opportunities by retrofitting the thermal environment such as rack positioning, containment implementation, and guided airflow control. A series of numerical simulations are performed using the validated numerical model and KPIs are calculated for each design. Numerical simulations demonstrate that the efficiency of an existing data center can be enhanced by up to 75% using the presented approach, although the improvement varied significantly with the specific KPIs. The computational approach proposed here can be readily generalized to guide the efficiency assessment and improvement of existing data centers.

## Introduction

The rapid advancement and global competition in artificial intelligence (AI) services have significantly increased the energy demand from data centers. In response, the European Commission adopted the Energy Efficiency Directive (EED) on September 13, 2023. This directive mandates that data centers in Europe with a power demand exceeding 500 kW must report key performance indicators (KPIs) based on data collected during the reporting period^1^. Consequently, this directive is poised to significantly impact data center operations and highlights the critical need for a systematic approach to assess and enhance the energy efficiency of existing data centers systematically.

Computational Fluid Dynamics (CFD) methods play a significant role in improving the efficiency of data centers by providing detailed insights into airflow distribution, thermal management and cooling optimization^2^. These models enable data center engineers to simulate and analyze various cooling strategies, identify hotspots, and optimize rack and server placement to reduce energy consumption while maintaining optimal thermal conditions outlined by standards. Integrating CFD simulations into the design and operation of data centers allows us to improve cooling efficiency, minimize recirculation zones, and reduce dependence on energy-intensive cooling systems. The application of CFD methods in data centers is not limited to traditional air-cooled systems^2–12^ but extends to liquid-cooled architectures^13–20^two-phase cooling solutions^21–23^and real-time dynamic workload allocation strategies^24–29^. Advanced CFD techniques enable high-resolution modeling of airflow, temperature distribution, and heat transfer within data centers under dynamic operating conditions. This detailed representation enhances the ability of energy management algorithms to adapt to real-time variations, such as changing IT loads or environmental conditions, thus improving their operational adaptability and control performance. Although commercial software packages are widely used for CFD modeling in data centers, the growing emphasis on sustainability is driving the adoption of open-source computational models^30–34^. The use of open-source CFD tools contributes to sustainability in multiple ways: it allows for flexible model development tailored to specific data center configurations and encourages transparency and reproducibility. Moreover, open-source tools can also be integrated with monitoring systems, facilitating the development of automated, data-driven optimization strategies that support energy efficiency and long-term sustainability goals. In this study, the computational model developed by the authors based on open-source libraries^35–37^ is employed for the simulations of airflow and thermal structures in the pilot data centers.

Efficient cooling is essential for the performance and longevity of IT components in air-cooled data centers. Furthermore, cooling energy consumption accounts for approximately 40% of the total energy usage in a data center, highlighting the critical need for optimized cooling solutions that align with KPIs. Consequently, energy efficiency has become an increasingly important priority, driving ongoing research and technological advancements in data center cooling^38–49^. The recently adopted EED emphasises the growing importance of improving energy efficiency, with a particular focus on reducing energy consumption and carbon emissions in data centers. In line with these objectives, the European Data Center Association (EUDCA) actively promotes the efficient management of data centers in guidelines. As an example, the EU Code of Conduct on Data Centre Energy Efficiency^1^ promotes best practices for the efficiency enhancement of existing data centers by highlighting the importance of CFD methods in the efficiency enhancement of data centers. Therefore, enhancing the energy efficiency of existing data centers has become a high priority, not only within Europe but also globally^50–52^.

Experimental data is essential for validating numerical models in the thermal modeling of data centers^6,12,35,36,53–55^. In this study, we employed two distinct experimental approaches for validating the numerical model at each pilot site. The first approach involved airflow and temperature measurements using external sensors, a widely used method in the literature^56–58^. The second approach collected IPMI data from server sensors, which has emerged as a practical approach for validating the numerical results. This combination of traditional and novel validation techniques enhances the robustness of our findings and demonstrates the versatility of our approach in the present data-driven validation.

Therefore, this study aims to address the growing need for improved energy efficiency in data centers by evaluating the performance of two European pilot data centers located in Denmark and Poland. We utilized CFD simulations to calculate KPIs related to airflow, thermal structure and cooling performance. The numerical model is validated using the experimental data collected from the pilot data centers, ensuring the accuracy of the simulations. The KPIs served as a basis for assessing both thermal and cooling efficiencies. Following the evaluation of the current performance, proposed improvements are implemented and analyzed numerically through a series of intermediate processes. The results, along with a detailed explanation of the improvement strategies, provide valuable insights for optimizing design of existing data centers.

## Methodology

### Descriptions of the pilot data centers

**Fig. 1:**
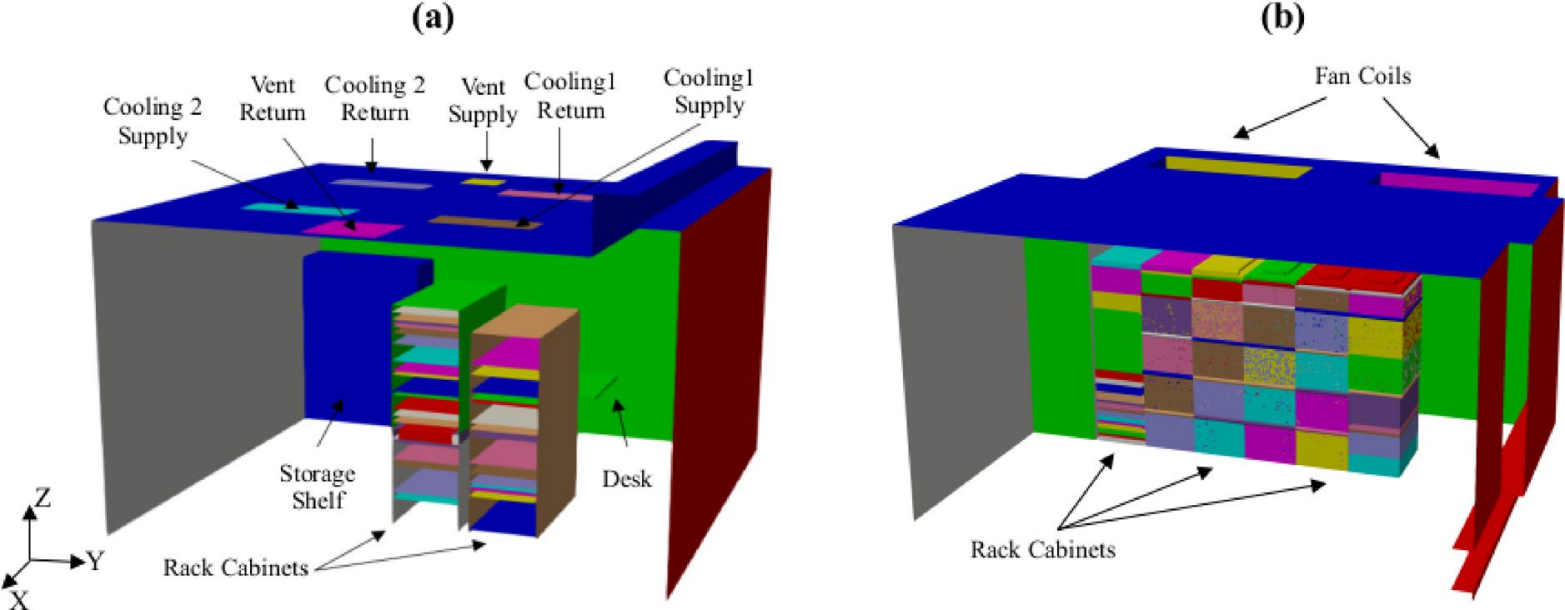
Three-dimensional views of pilot data centers: (**a**) PSNC and (**b**) AAU data centers.

The first data center considered in this study is a micro-data center located at the Poznan Supercomputing and Networking Center (PSNC). As shown in Fig. 1a, this open aisle data center, consisting of two rack cabinets, is cooled by ceiling-mounted air conditioners. The second data center shown in Fig. 1b, serves as a hub for research and education at Aalborg University (AAU) in Denmark. This data center is equipped with six rack cabinets and two fan coil units, utilizing an air-cooled system for efficient temperature regulation and reliable operation. Both the PSNC and AAU data centers serve as pilot data centers in the EU funded HEATWISE project^59^providing benchmarking cases for assessing and improving energy efficiency of server rooms at tertiary buildings.

**Fig. 2:**
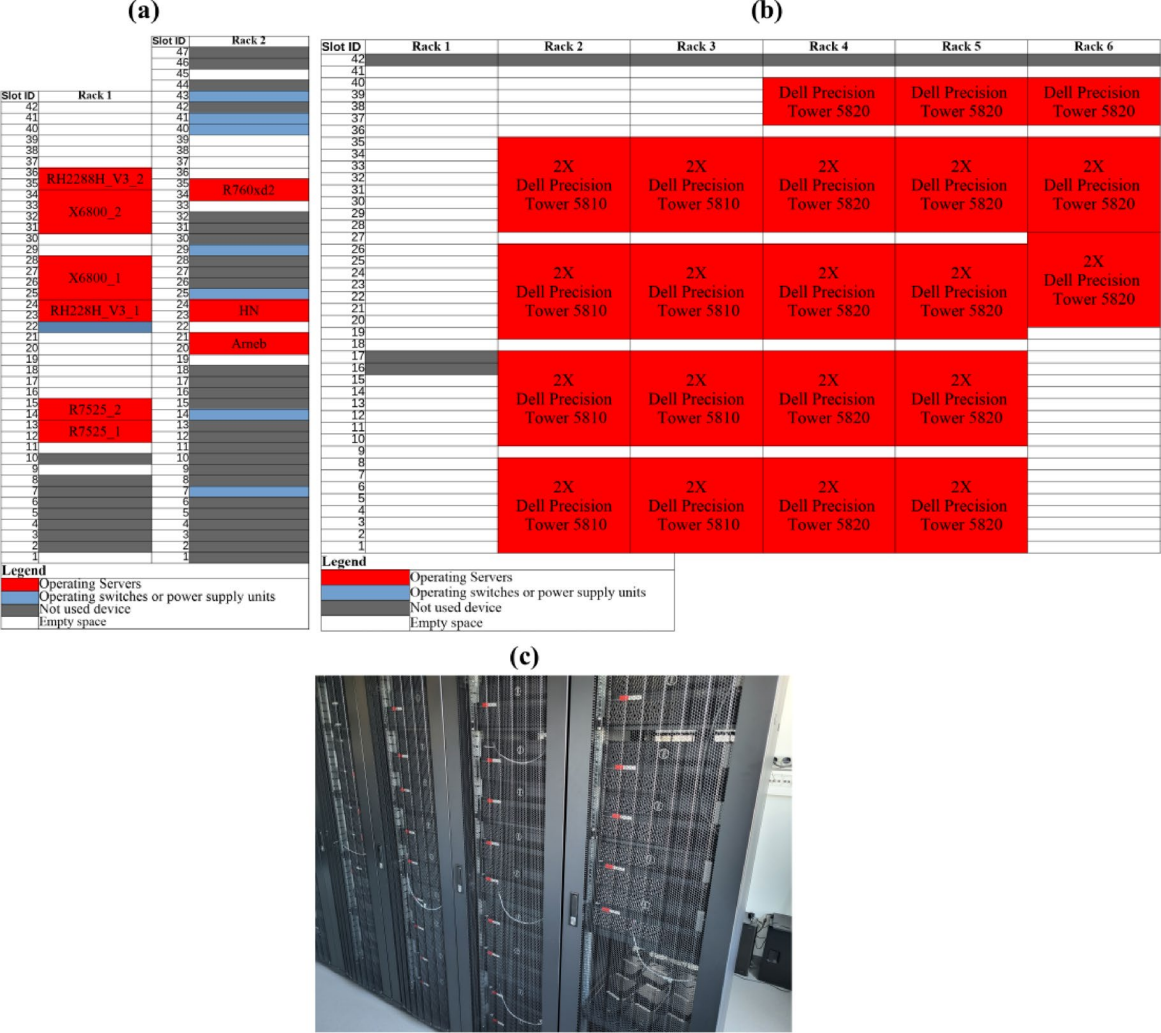
Rack layouts of (**a**) PSNC, (**b**) AAU data centers and (**c**) physical racks of AAU data center.

Figure 2 shows layouts of the standard 42U and 47U rack cabinets deployed in the data centers along with the snapshot of the racks in AAU data center. The PSNC data center houses 9 active servers across 2 racks, while the AAU data center operates 21 active servers across 6 racks. While all servers provide standard airflow direction, the Arneb server in the PSNC data center (Fig. 2a) has a different chassis construction and is designed to be cooled by an airflow rotated 90 degrees horizontally compared to the other servers, which will be discussed further during the validation of the numerical model. Power consumption and flow rates of servers were measured during the experimental studies to construct and validate the numerical model under the same workloads as in the experimental studies.

### Experimental studies at the pilot data centers

Two distinct experimental campaigns were carried out concurrently at the pilot data centers to validate the numerical model with the experimental measurements. The experimental campaign conducted at the AAU data center consisted of measuring airflow velocities and temperatures. Airflow velocities were measured using sensors 16 calibrated Dantec Dynamics model 54R103 anemometers, which have a measurement range of 0–5 m/s and an uncertainty of +/- 5%. Temperature measurements were conducted using calibrated PT100 temperature sensors located at the inlet and outlet of each server, as well as at the strategic locations in the room where recirculation effects are observed. The entire temperature measurement setup had an uncertainty of 0.21 °C. The power consumption was recorded at the rack level using Power Distribution Units (PDUs) in the form of a Norma D5255S with a maximum uncertainty of +/- 1.25%. Since all servers were tower-types, their individual power consumption was determined from evenly splitting the rack-level data among the servers. As the servers were similar and kept at similar CPU loads, this is, though, not expected to add significant uncertainty in the measurements. The measured power consumption and flow rate for each rack are detailed in Table 1.

**Fig. 3:**
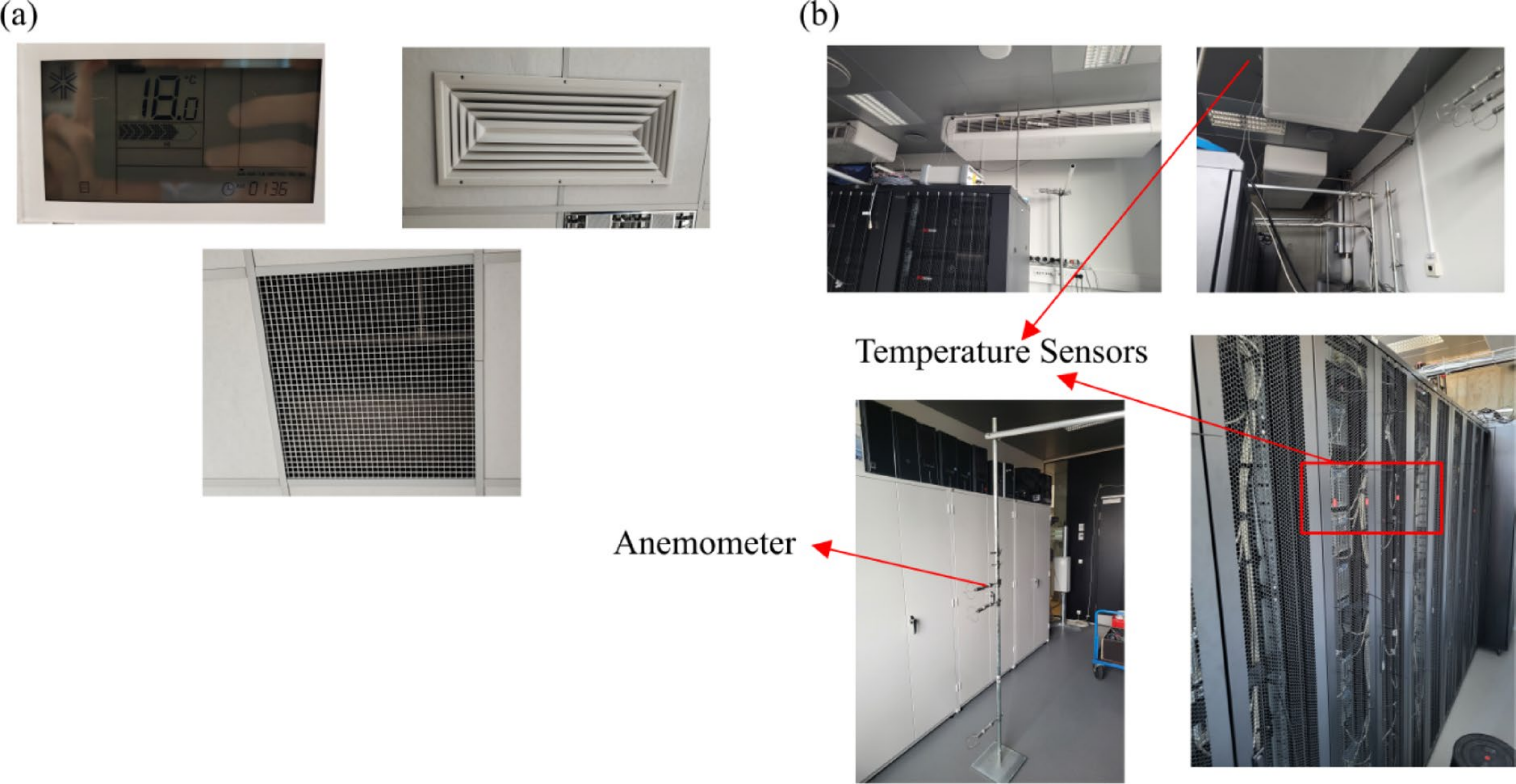
Experimental setup of (**a**) PSNC and (**b**) AAU data centers.

At the PSNC data center, power consumptions and temperatures were collected from embedded server sensors, a practical and straightforward method for simultaneously gathering IT and thermal data at the server level. In this context, the IPMI data was collected over two days with a 10-minute sampling interval to obtain adequate data for the calculation of time-averaged values. The dataset collected via IPMI includes inlet and outlet temperatures, server loads, and fan speeds for some of the servers. Additional thermal parameters were measured using external sensors to ensure comprehensive validation of the numerical model. Specifically, Serverscheck Wireless Sensors were employed to record inlet and outlet temperatures of the servers, with the combined standard uncertainty of the measurement at the level of 0.35 °C. The HTemp-1 Wire 3 m Sensors with the measurement uncertainty of 0.52 °C were used to monitor the temperature of the cooling and ventilation system. To capture airflow velocity, Omron D6F-W04A1 Sensors were utilized, for which the measurement uncertainty was equal to 2.9%. Furthermore, supply and return temperatures of the cooling and ventilation units, as well as the power consumption of the cooling system, were recorded to accurately represent the thermal conditions in the numerical model. Figure 3 shows the experimental setup including locations of temperature sensors and anemometers.

**Table 1:** Layouts of PSNC and AAU data centers.

Data Center	Rack	Server Position	Height (U)	Power Consumption (W)	Flow Rate (m^3^/s)
PSNC	1	12	2	304.4	0.05
	1	14	2	128.18	0.05
	1	23	2	308.07	0.04
	1	25	4	968.96	0.1
	1	31	4	982.52	0.13
	1	35	2	321.04	0.05
	2	20	2	668.4	0.04
	2	23	2	136.74	0.02
	2	34	2	265.3	0.03
AAU	2	1	8	250	0.075
	2	10	8	250	0.075
	2	19	8	250	0.075
	2	28	8	250	0.075
	3	1	8	73	0.01275
	3	10	8	73	0.01275
	3	19	8	73	0.01275
	3	28	8	73	0.01275
	4	1	8	73	0.01425
	4	10	8	73	0.01425
	4	19	8	73	0.01425
	4	28	8	73	0.01425
	4	37	4	73	0.01425
	5	1	8	73	0.0165
	5	10	8	73	0.0165
	5	19	8	73	0.0165
	5	28	8	73	0.0165
	5	37	4	73	0.0165
	6	20	8	73	0.01125
	6	28	8	73	0.01125
	6	37	4	73	0.01125

### CFD modeling of the pilot data centers

Turbulent flow and energy distribution within the data center can be represented by the following continuity, momentum and energy equations:

1 $$\:\frac{\partial\:\rho\:}{\partial\:t}+\frac{\partial\:}{\partial\:{x}_{i}}\left(\rho\:{u}_{i}\right)=0$$

2 $$\:\frac{\partial\:\left(\rho\:{u}_{i}\right)}{\partial\:t}+\frac{\partial\:}{\partial\:{x}_{i}}\left(\rho\:{u}_{i}{u}_{j}\right)=\frac{-\partial\:p}{\partial\:{x}_{i}}+\frac{\partial\:}{\partial\:{x}_{j}}\left[\left(\mu\:+{\mu\:}_{t}\right)\left(\frac{\partial\:{u}_{i}}{\partial\:{x}_{j}}+\frac{\partial\:{u}_{j}}{\partial\:{x}_{i}}\right)+\rho\:{g}_{i}+{S}_{i}\right]$$

3 $$\:\frac{\partial\:\left(\rho\:h\right)}{\partial\:t}+\frac{\partial\:}{\partial\:{x}_{j}}\left(\rho\:{u}_{j}h\right)=\frac{\partial\:}{\partial\:{x}_{j}}\left[\left(\mu\:+{\mu\:}_{t}\right)\frac{\partial\:h}{\partial\:{x}_{i}}\right]$$

Where $\:\rho\:$ is the density of the fluid, $\:{u}_{i}$ is the mean velocity component in the i-direction, $\:t$ is the time, $\:p$ is the pressure, $\:{x}_{i}$ and $\:{x}_{j}$ are the Cartesian coordinates, $\:{g}_{i}$ is the gravitational acceleration in the i-direction, $\:h$ is the Favre-averaged enthalpy, $\:{S}_{i}$is the momentum source to set a directed flow through the server, $\:\mu\:$ and $\:{\mu\:}_{t}$ are molecular and turbulent viscosities, respectively. Molecular viscosity is calculated using the Sutherland viscosity model considering temperature effects and turbulent viscosity can be calculated from the following equation:

4 $$\:\nu\:=\frac{{\alpha\:}_{1}k}{max\left({\alpha\:}_{1}\omega\:,{SF}_{2}\right)}$$

The k-ω Shear Stress Transport (SST) turbulence closure model is employed to account for adverse pressure gradients and boundary layer effects near the walls. The turbulence kinetic energy and specific rate of dissipation are determined from the solutions of the following transport equations:

5 $$\:\frac{\partial\:k}{\partial\:t}+{u}_{i}\frac{\partial\:k}{\partial\:{x}_{i}}=\frac{\partial\:}{\partial\:{x}_{j}}\left[\left(\nu\:+{{\sigma\:}_{k}\nu\:}_{t}\right)\frac{\partial\:k}{\partial\:{x}_{i}}+P+\beta\:k\omega\:\right]$$

6 $$\:\frac{\partial\:\omega\:}{\partial\:t}+{u}_{i}\frac{\partial\:\omega\:}{\partial\:{x}_{i}}=\frac{\partial\:}{\partial\:{x}_{j}}\left[\left(\nu\:+{{\sigma\:}_{\omega\:}\nu\:}_{t}\right)\frac{\partial\:\omega\:}{\partial\:{x}_{i}}+\alpha\:{S}^{2}+\beta\:{\omega\:}^{2}+2\left(1-F\right)\frac{{\sigma\:}_{\omega\:2}}{\omega\:}\frac{\partial\:k}{\partial\:{x}_{i}}\frac{\partial\:\omega\:}{\partial\:{x}_{i}}\right]$$

Coefficients that appear in the turbulence equations can be found in the literature^60^.

Flow and thermal fields in the data centers are simulated based on the numerical solution of the above equations using a custom solver dataCenterDST^35–37^which is specialized in the thermal simulations of air-cooled data centers. The dataCenterDST is a customized OpenFOAM solver developed by the authors to perform thermal simulations of data centers, considering the specific geometry, rack layout and operational conditions of the facility in a single framework. The dataCenterDST mainly creates server racks, applies momentum and energy sources to the servers using an open-box server modelling approach and considers the pressure drop experienced in the turbulent flow through the servers using the Darcy-Forchheimer porosity model^61^.

The cooling and ventilation units are modelled using the black-box approach, where inlet and outlet boundary conditions are specified as Dirichlet type boundary conditions in the numerical model. At the outlet of the cooling units, boundary conditions corresponding to a fixed flow rate for velocity, a gauge pressure condition for pressure and a zero-gradient condition for temperature are applied. Buoyancy effects are modeled using the buoyantPimpleFoam solver, which numerically solves the compressible Navier–Stokes equations with temperature-dependent air density calculated via the ideal gas law. This allows for accurate simulation of buoyancy-driven flow, thermal stratification, and natural convection in a data center.

At the inlet of the cooling units, a reverse flow-tolerant condition for velocity, a fixed gradient condition for pressure, and a fixed temperature value are specified. No-slip boundary conditions are imposed for velocity on the walls.

**Fig. 4:**
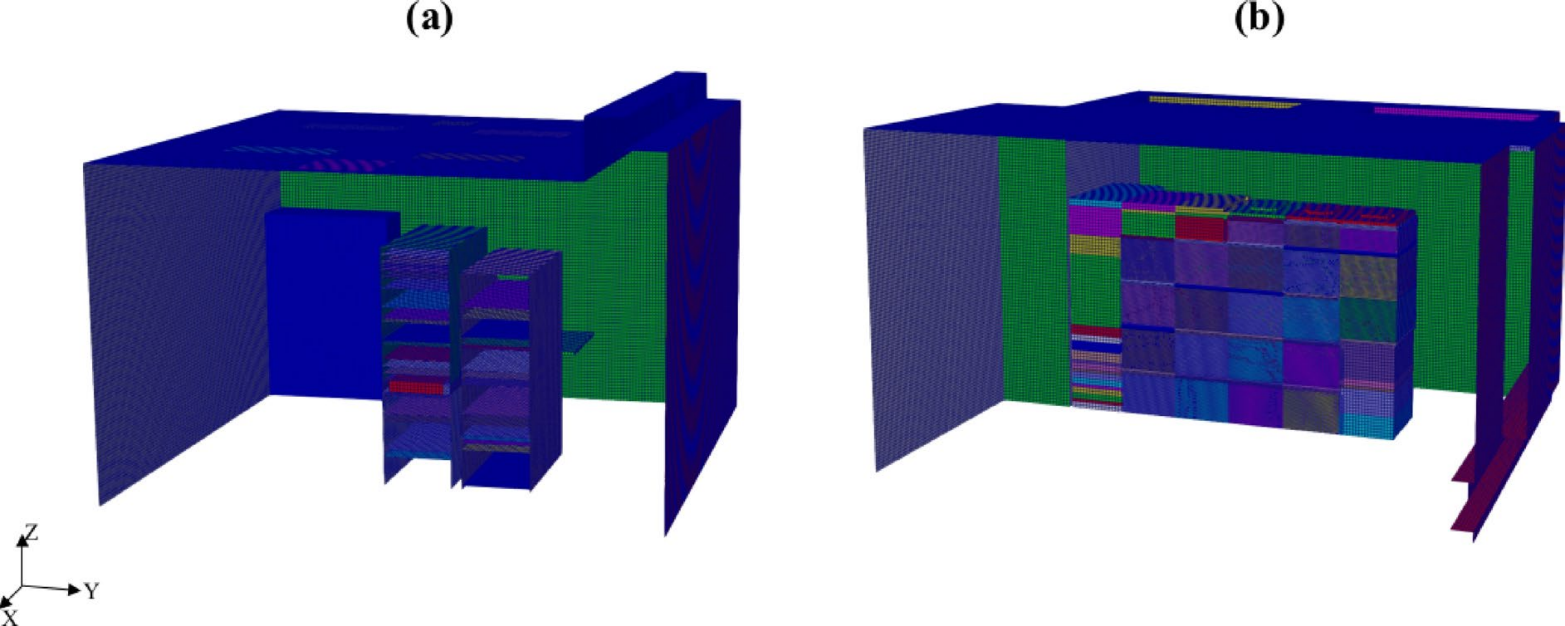
Computational meshes for (**a**) PSNC and (**b**) AAU data centers.

Initially, a structured base mesh was generated to represent the room geometry. Subsequently, a mesh refinement algorithm was employed to locally refine the grid around the racks and servers, ensuring accurate resolution of the heat-generating components within the computational domain. This algorithm, which is commonly used in CFD applications, generates a hexahedral-dominant mesh with local refinement capabilities, ensuring a balance between computational efficiency and accuracy. To accurately capture the geometry and thermal behavior near the racks and servers, a maximum of three levels of mesh refinement was applied in these regions. The mesh was generated in parallel to enhance performance. The resulting meshes depicted in Fig. 4 are hexahedral dominant ensuring a balance between computational efficiency and accuracy.

A mesh independent study is performed systematically for each data center. Table 2 presents the total number of computational cells used for different mesh resolutions across two data center configurations ranging from coarse (Mesh1) to fine (Mesh5) grids.

**Table 2:** Number of cells for different mesh resolutions.

Data Center	Mesh1	Mesh2	Mesh3	Mesh4	Mesh5
PSNC	1,003,784	1,314,626	1,652,180	1,824,308	2,042,524
AUU	634,112	821,213	1,066,351	1,118,432	1,312,104

**Fig. 5:**
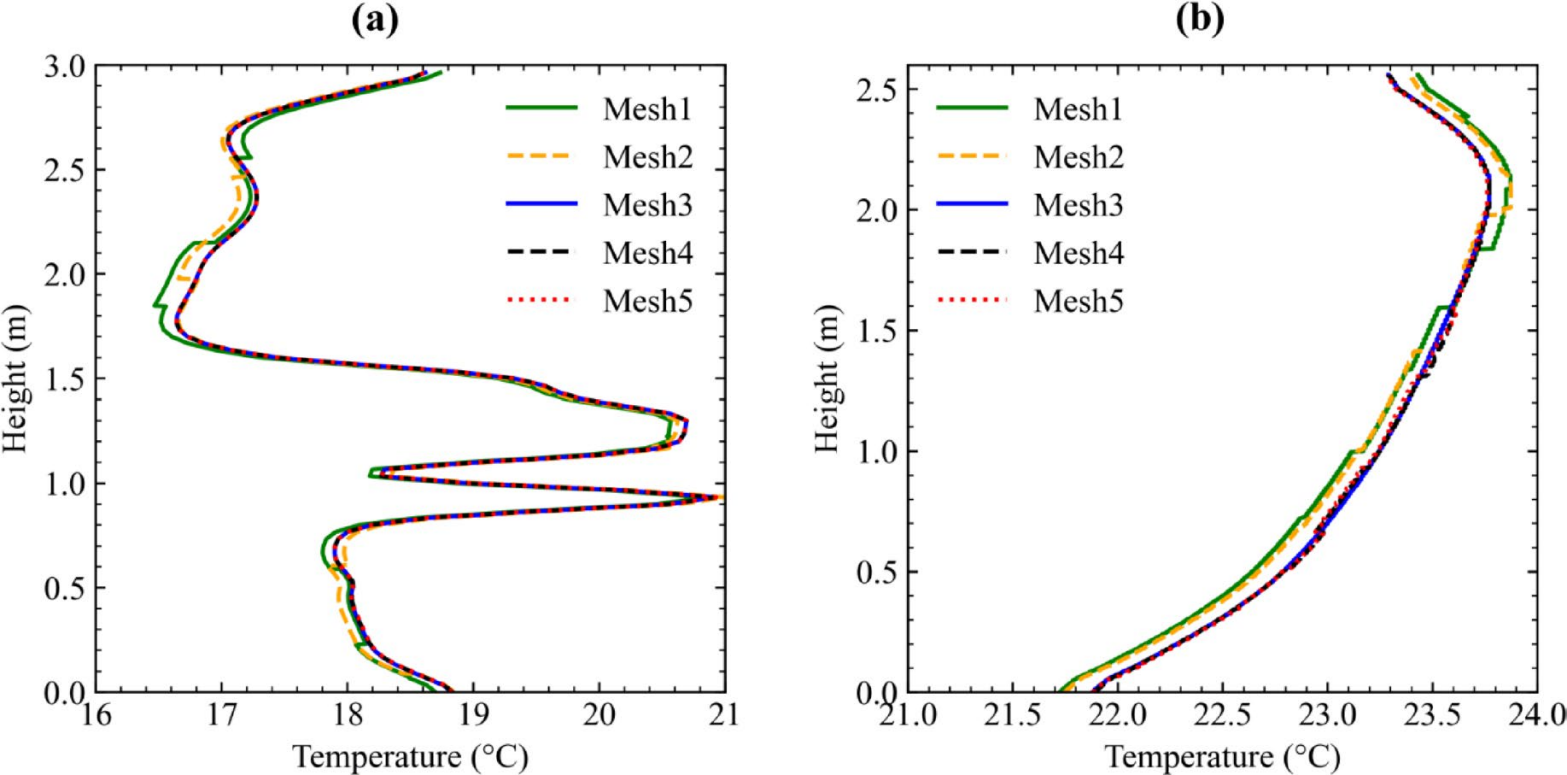
Mesh independence study of (**a**) PSNC and (**b**) AAU data centers.

Vertical temperature profiles were extracted at a location of 0.4 m behind the center of the rack outlets and compared at representative rack positions for each mesh. Figure 5a and b demonstrate that temperature distributions become increasingly consistent with mesh refinement. Notably, the results from Mesh3 show good agreement with those from finer meshes (Mesh4 to Mesh5), indicating that further refinement yields negligible changes in temperature profiles. Therefore, Mesh3 was selected for both data center configurations as it offers a good balance between computational cost and accuracy.

Key statistics of the meshes are listed in Table 3 demonstrating that the mesh features are suitable for the accurate calculation of fluxes on cell faces. Numerical simulations are performed with parallel computing on the computational resources provided by the HEATWISE project^59^.

**Table 3:** Statistics of the computational meshes generated for pilot data centers.

Data center	Number of cells	Max. Skewness	Max Non-orthogonality	Min y^+^	Max y^+^	Min. Volume	Max. Volume
PSNC	1,652,180	0.00	0.00	1.73	9.16	3.57 × 10^−5^	3.57 × 10^−5^
AUU	1,066,351	2.89	62.73	1.13	9.84	5.99 × 10^−7^	3.4 × 10^−4^

### KPIs for thermal and cooling efficiencies

An effective thermal design is essential not only for the optimal performance and reliable operation of IT infrastructure, but also for reducing cooling energy consumption in data centers. Thermal and cooling efficiencies of a data center design can be assessed and reported according to various KPIs. Key effects that reduce the efficiency of a data center, such as hot air recirculation and cold air bypass, can be measured as deviations from the recommended and allowable temperatures (Table 4) outlined by the ASHRAE standard^62^ In this context, the.

$\:{T}^{min-rec}$ and $\:{T}^{max-rec}$ refer to the upper and lower bounds of the recommended inlet temperature range, respectively, which are intended to ensure optimal equipment reliability and energy efficiency. On the other hand, the $\:{T}^{min-all}$ and $\:{T}^{max-all}$ indicate the broader allowable temperature range, which defines the safe operational limits for IT equipment without immediate risk of failure, but potentially with reduced lifespan or efficiency.

**Table 4:** Recommended and allowable air temperatures by standards.

$\:{T}^{max-rec}$ (°C)	$\:{T}^{min-rec}$ (°C)	$\:{T}^{max-all}$ (°C)	$\:{T}^{min-all}$ (°C)
27	18	32	15

Server inlet temperatures ($\:{T}_{in}$) are often observed to be higher than the cooling supply temperature ($\:{T}_{sup}$). Conversely, the air temperature at the inlet of the cooling unit, also known as the return temperature, is often lower than the server exhaust temperature due to the turbulence and buoyancy effects in the flow and thermal structures, respectively. The Rack Cooling Index (RCI)^35^ measures cooling efficiency of a data center by comparing server inlet temperatures with maximum and minimum recommended values:

7 $$\:{RCI}_{HI}=\left[1-\frac{\sum\:_{i=0}^{n}\left({T}_{in}-{T}_{max-rec}\right)}{n\times\:\left({T}_{max-all}-{T}_{max-rec}\right)}\right]\times\:100\%$$

8 $$\:{RCI}_{LO}=1-\left[\frac{\sum\:_{i=0}^{n}\left({T}_{min-rec}-{T}_{in}\right)}{n\times\:\left({T}_{min-rec}-{T}_{min-all}\right)}\right]\times\:100\%$$

Another metric for assessing cooling performance is the Return Heat Index (RHI), which compares total IT heat dissipation to the heat removed by the cooling system. Higher RHI values indicate better cooling efficiency and reliability^35^.

9 $$\:\frac{Total\:heat\:dissipation}{Total\:heat\:dissipation+Rise\:in\:the\:enthalpy\:of\:the\:air}$$

The Return Temperature Index (RTI)^63^ compares return and supply air temperatures of the cooling system, normalized by the expected temperature difference:

10 $$\:RTI=\left[1-\frac{{T}_{return}-{T}_{sup}}{{\varDelta\:T}_{equip}}\right]$$

Kuzay et al. (2023) proposed the Recirculation Index (RI) to assess the impact of recirculation on the rack^37^:

11 $$\:RI=\left[1-\frac{{T}_{rackInlet,mean}-{T}_{sup}}{{T}_{sup}}\right]$$

Here, the $\:{T}_{rackInlet,mean}$ is the average temperature calculated at the inlet of the rack. Allowable and target values of the KPIs are listed in Table 5 to represent ideal conditions with no hot air recirculation and cold air bypass within the data center. Consequently, the performance of a data center design can be classified according to the ranges given in Table 5.

**Table 5:** Targets and allowable ranges of the KPIs.

Index	Target	Allowable Range
RCI_HI_^36^	100%	≥ 96%
RCI_LO_^36^	100%	≥ 96%
RTI^64^	100%	95–105%
RHI^36^	1	> 0.8
RI^38^	100%	100%

**Fig. 6:**
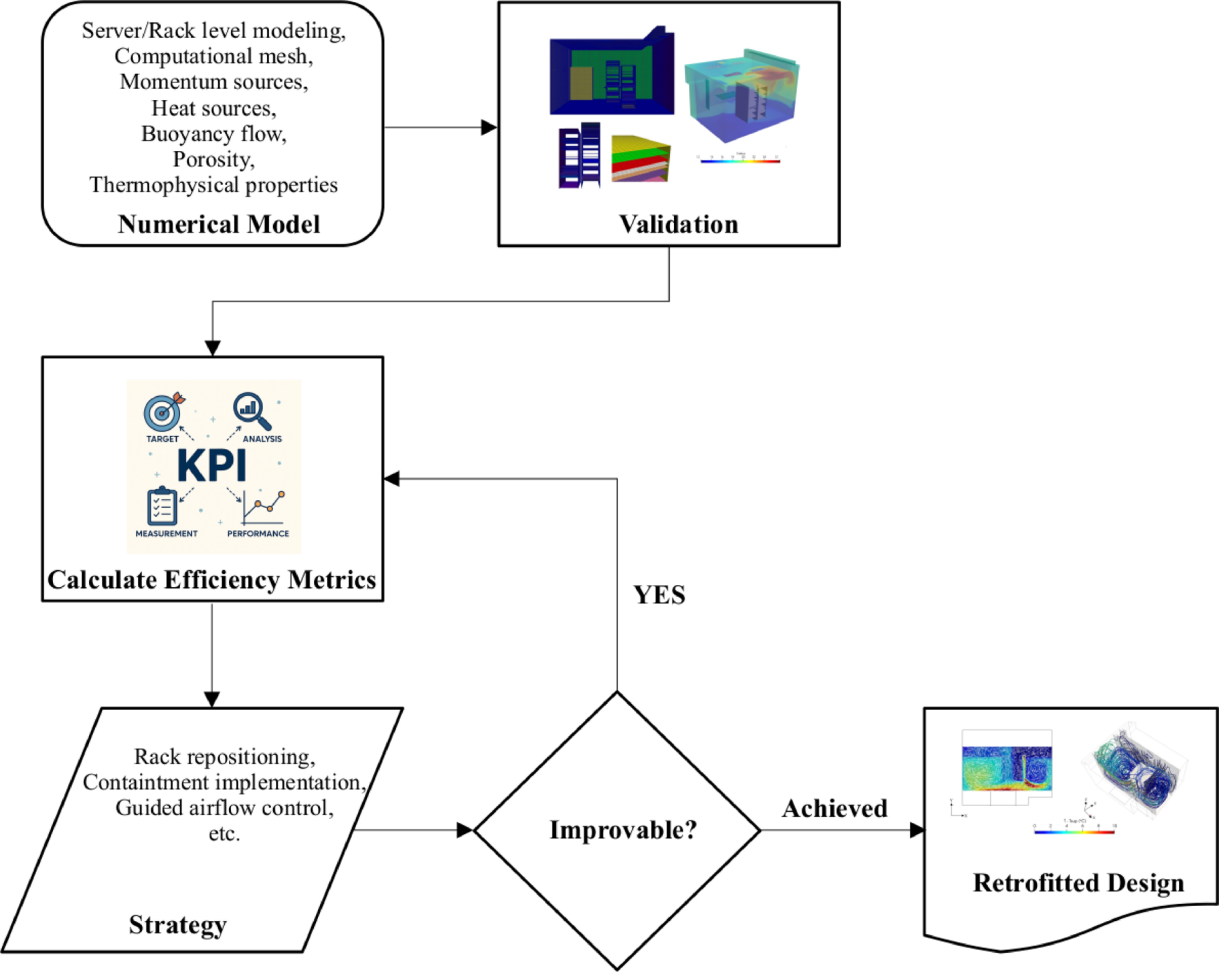
Flow chart for the KPI-informed enhancement approach.

In this study, multiple physical retrofitting strategies were evaluated, including repositioning and shifting of server racks, implementation of hot and cold aisle containment, and narrowing of the airflow domain to improve flow guidance. Each configuration was simulated using the validated CFD model and assessed based on a set of cooling and thermal KPIs. The configuration presented in the manuscript represents the most efficient solution identified through this comparative analysis. As the simulations were guided by KPI values, this approach is referred to as a KPI-informed retrofitting strategy. Figure 6 presents schematic representation of the integrated working principle underlying the retrofitting strategy, which was formulated through a CFD based optimization framework. The flow chart represents the iterative process in which CFD simulations are employed to assess thermal performance, while KPI metrics provide quantifiable benchmarks to guide successive design refinements, ensuring that the retrofitted design achieves optimal thermal management.

## Results and discussions

### Validation of the numerical model with the experimental data

In the numerical model, the power consumption data collected from the experimental studies was applied implicitly as a heat source within the servers using a source term. The Darcy and Forchheimer coefficients were calculated for the porosity value of 0.5 to represent the flow resistance caused by the internal components of the servers^61,64^. The measured flow data presented in Table 1 was incorporated as a momentum source in the corresponding server models to ensure that the air flow rates of servers match the flow rates measured in the experimental studies.

**Fig. 7:**
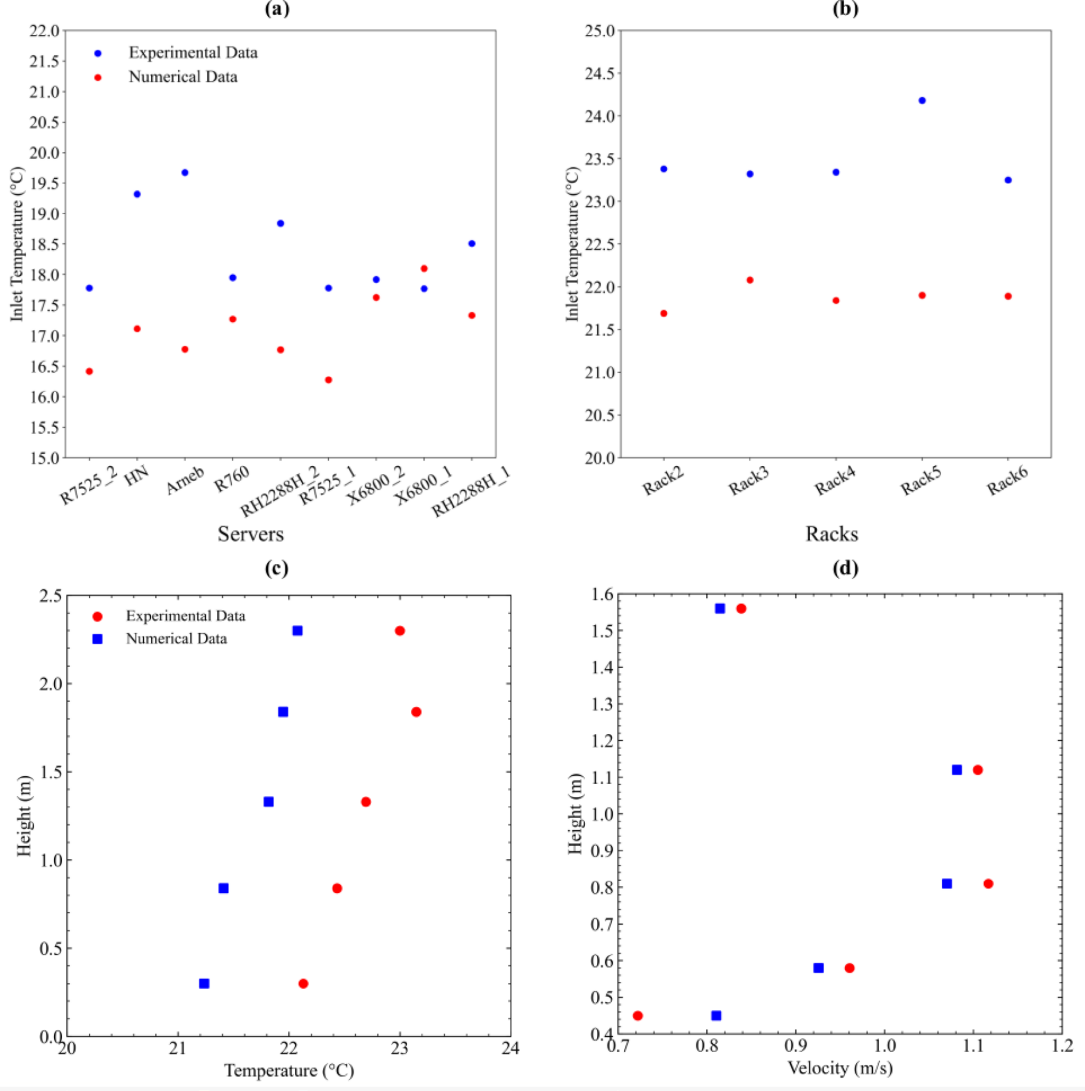
Comparison between measured and simulated mean inlet temperatures at the (**a**) PSNC and (**b**) AAU data centers, along with a comparison of vertical (**c**) temperature and (**d**) velocity profiles within the interior of the AAU data center.

Figure 7 compares mean inlet temperatures of servers obtained from experimental and numerical results. Average absolute temperature differences between numerical and experimental data were calculated as 1.3 °C and 1.6 °C at PSNC and AAU data centers, respectively, which may be associated with the measurement accuracy of the sensors, numerical errors arising from the discretization of partial differential equations and computational mesh size in the present numerical implementation. As can be seen in Fig. 7a, the discrepancy around the Arneb server increases due to fact that this server provides an airflow rotated 90 degrees horizontally compared to the other servers and the airflow around the Arneb server was influenced by the nearby servers and wall interactions, leading to variations in the inlet temperature distribution. As the Arneb server was positioned close to other servers and walls, localized turbulence and recirculation zones, which resulted in unexpected cooling effects and significantly influenced prediction of the inlet temperatures. The vertical profile for velocity was placed 0.7 m in front of the inlet of Rack 6, while the vertical profile for temperature was positioned 0.7 m behind Rack 6. Figure 7c and d present the comparisons between experimental data obtained from temperature and velocity sensors along these two different vertical profiles inside the AAU interior room and corresponding numerical simulation results.

### Assessment of thermal and cooling efficiencies of pilot data centers

**Fig. 8:**
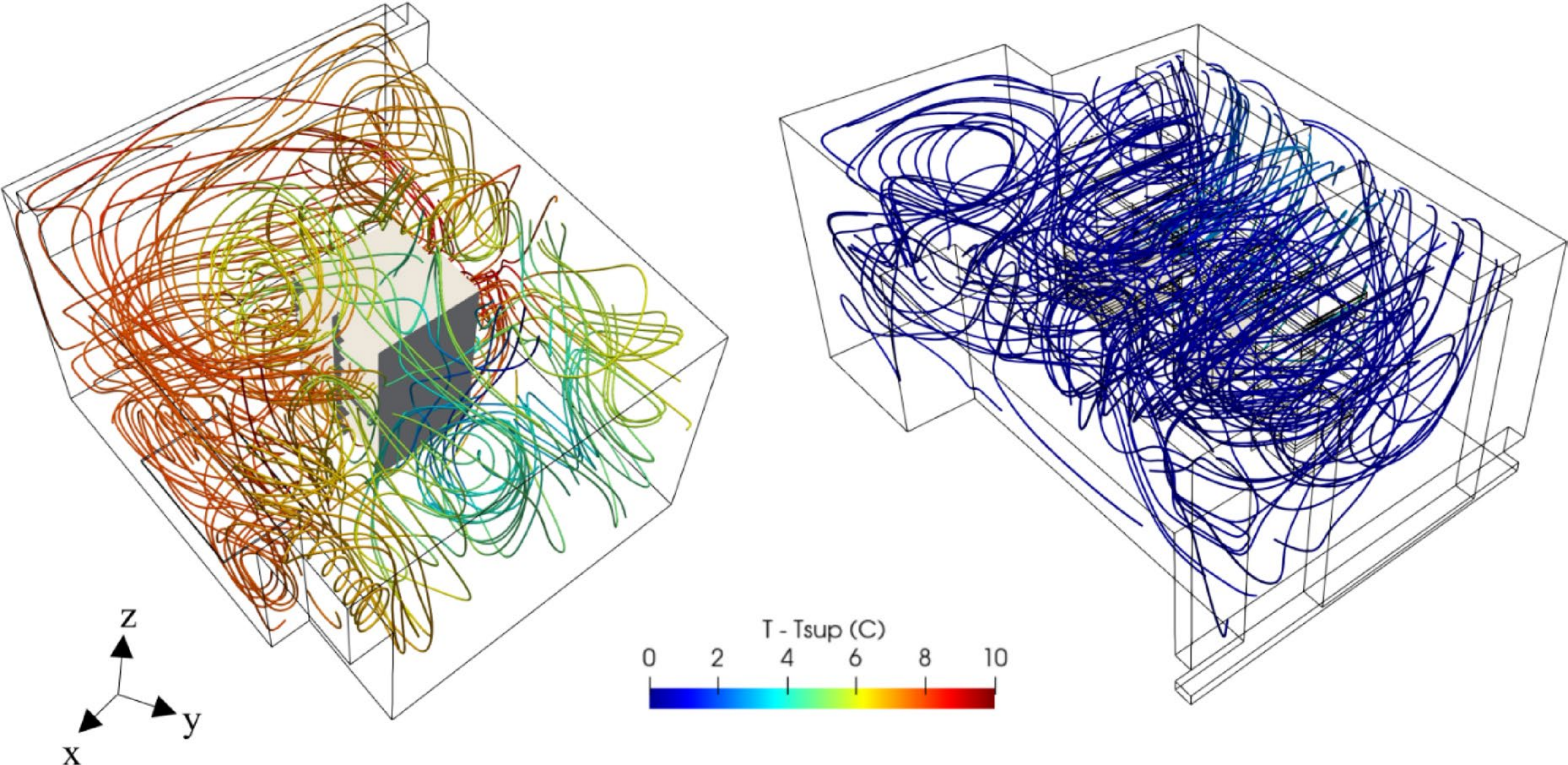
Velocity vectors and temperature fields at (**a**) PSNC and (**b**) AAU data centers.

The validated Computational Fluid Dynamics (CFD) simulation results were used to calculate relevant KPIs for the assessment of current thermal and cooling efficiencies of data centers. These KPIs were systematically analyzed and presented in Table 6, providing valuable insights into the operational performance of both data centers. Specifically, the KPIs calculated for the cooling units in the PSNC data center significantly deviated from the optimal ranges listed in Table 5. This deviation was primarily due to the suboptimal positioning of the ceiling mounted ventilation units relative to the main cooling units (Fig. 1). Additionally, as seen in Fig. 8a, hot and cold air mixing in the open aisle PSNC data center significantly impacted the RCI. Furthermore, the operation of two cooling units with different supply temperatures contributed to the RCI deviation. The analysis revealed insufficient cold air delivery to the servers, while heated exhaust air was recirculating toward the inlets, which substantially increased the heat load on the cooling units. Therefore, efforts focused on identifying geometric improvements to minimize mixing of hot and cold air.

**Table 6:** KPIs for PSNC and AAU data centers.

Data Center	$\:{RCI}_{HI}$	$\:{RCI}_{LO}$	$\:RTI$	$\:RHI$	$\:RI$
PSNC	301.27	64.55	275.20	0.32	69.52
AAU	205.80	174.20	46.11	0.78	97.21

The AAU Data Center, as shown in Fig. 8b, exhibited relatively good thermal performance, with the RI and RHI values within the allowable ranges listed in Table 2. However, the RTI metric indicated areas for improvement, particularly in mitigating hot air recirculation. The vortices observed near the corners indicated localized airflow recirculation. Additionally, cold air bypass around the racks could lead to reduced cooling efficiency. These observations suggest new retrofitting alternatives to suppress hot air recirculation and cold air bypass by directing airflow using vanes.

### Retrofitting of the PSNC data center

**Fig. 9:**
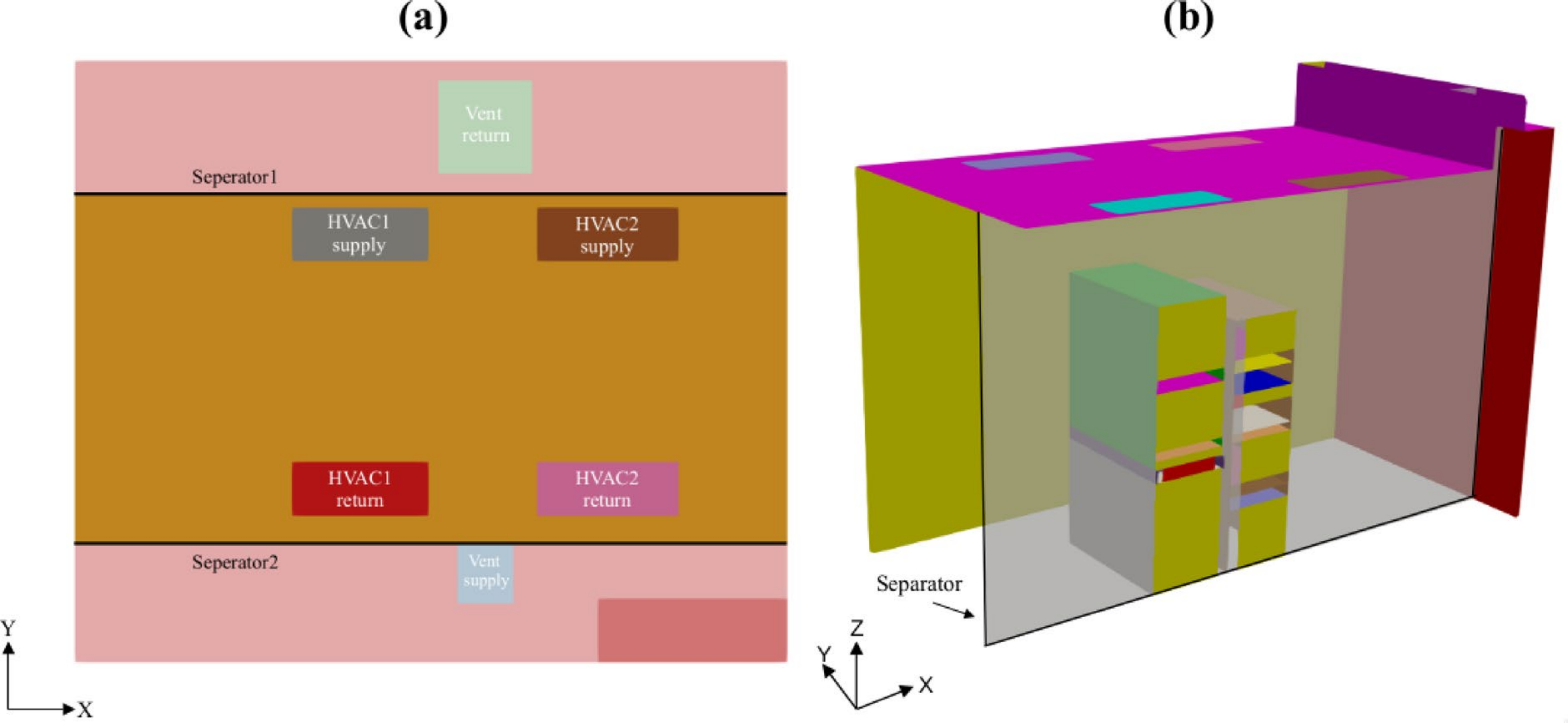
(**a**) Top and (**b**) three-dimensional views of the retrofitted design for PSNC data center.

The optimization process for the PSNC data center involved developing improvement stages by evaluating the impact of the design change on the thermal structure and KPIs. As depicted in Fig. 9a, ceiling-mounted ventilation units were removed from the design and airflow management separators were used to minimize thermal mixing between hot and cold air and enhance cooling efficiency. These separators were strategically positioned to enforce a hot and cold aisle containment system, effectively preventing cold and hot air mixing and optimizing thermal management within the data center. As shown in Fig. 9b, front and rear surfaces of inactive servers have been sealed to prevent air leakage, which helps maintain efficient airflow management, reduces cooling losses and improves overall thermal performance.

The relatively low KPIs observed in the PSNC data center can be attributed to the inlet temperatures being close to the minimum allowable limits recommended by ASHRAE depending on the supply temperature. During the retrofitting phase, the supply temperature was deliberately kept constant to ensure accurate validation of the implemented changes. The results demonstrate that the retrofitted design achieved 50% improvement in the RHI and 31% enhancement in the RI (Table 7). These substantial efficiency gains highlight the effectiveness of the retrofitted design as a highly viable solution for enhancing sustainable cooling performance in data center operation.

**Table 7:** KPIs for original and retrofitted designs along with the efficiency improvements.

KPI	Original	Retrofitted	Improvement (%)
RCI_HI_	301.27	357.52	−18.67
RCI_LO_	64.55	29.20	−54.76
RTI	275.20	128.32	53.33
RHI	0.32	0.48	50.00
RI	0.48	91.19	31.17

**Fig. 10:**
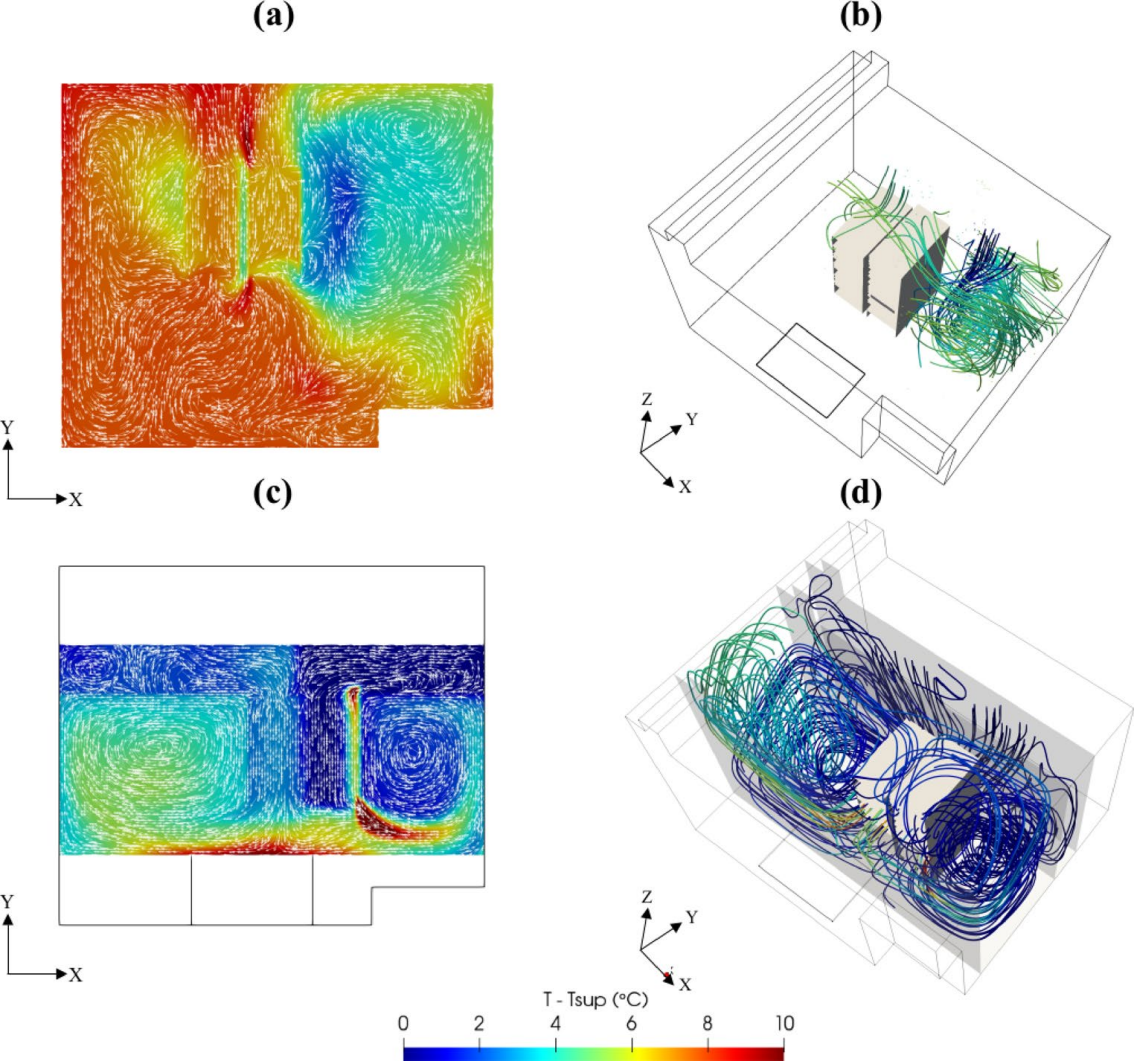
(**a**) Temperature distribution on the plane at *z* = 1.00 m (**b**) three-dimensional flow and thermal structures of original design and (**c**) Temperature distribution on the plane at *z* = 1.00 m and (**d**) three-dimensional flow and thermal structures of retrofitted design of PSNC data center.

The retrofitted design effectively mitigates the risk of hotspot formation while preventing the leakage of cold air into non-essential areas. By establishing more uniform thermal conditions, it enhances cooling efficiency, reduces energy consumption, and optimizes overall system performance. As illustrated in Fig. 10, the implementation of the retrofitted design has led to a significant reduction in the average temperature across the PSNC data center.

### Retrofitting of the AAU data center

A systematic investigation was conducted to evaluate various preliminary design modifications for the AAU data center. The effectiveness of these modifications was quantitatively assessed using KPIs to determine the most optimized configuration for airflow management and thermal performance. The primary objectives are to enhance cooling efficiency, minimize thermal mixing, and establish a more controlled and uniform thermal environment within the data center.

**Fig. 11:**
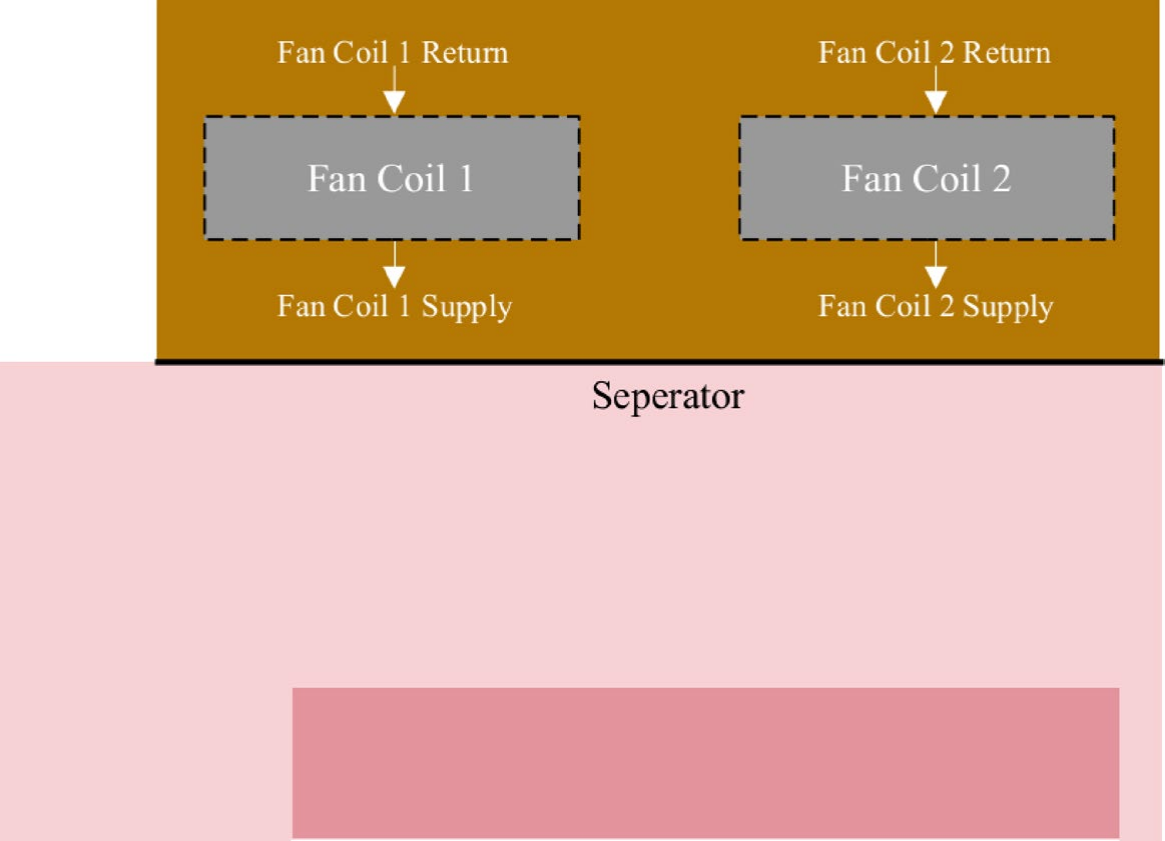
Top view of the retrofitted design.

**Fig. 12:**
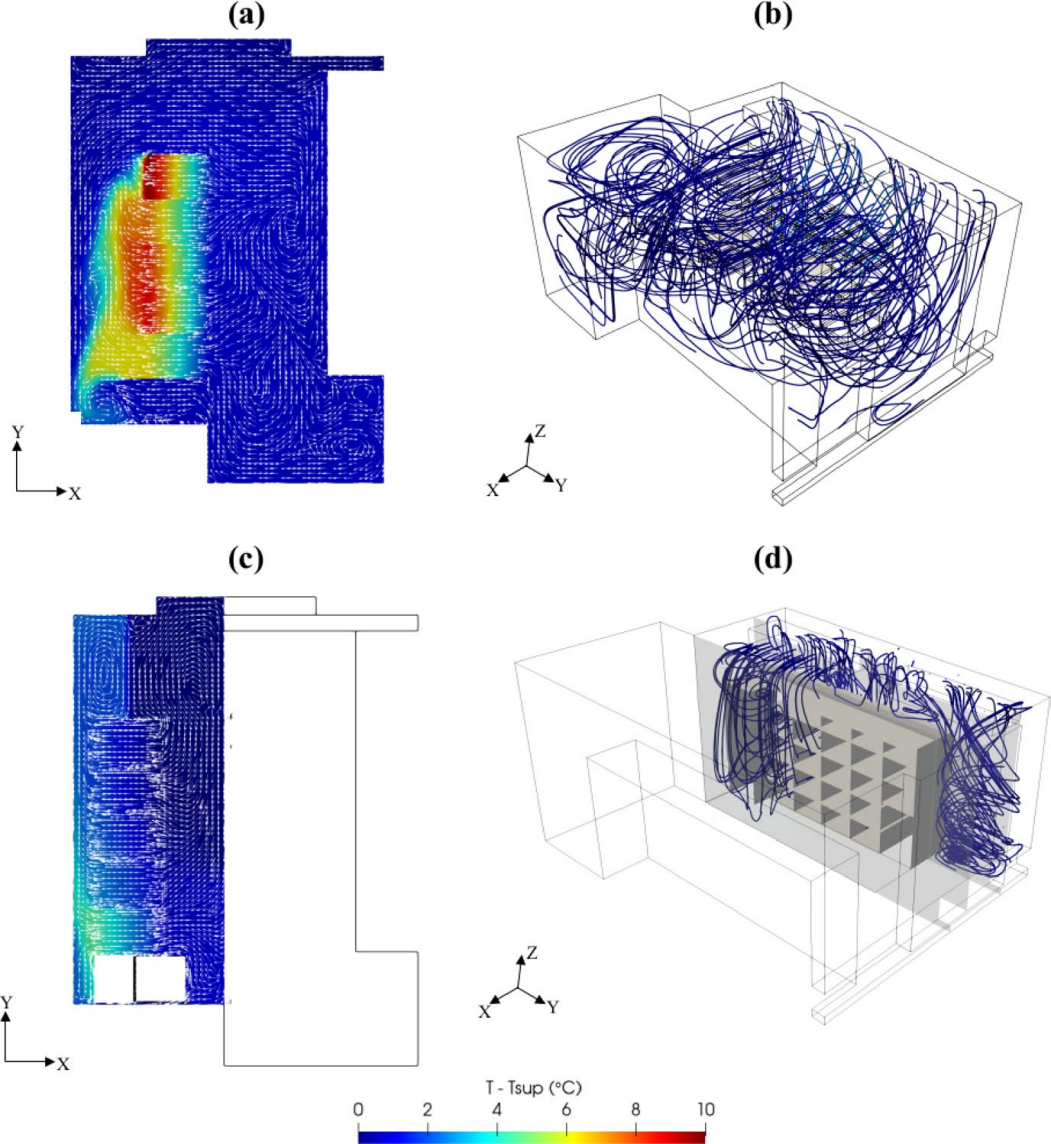
(**a**) Temperature distribution at z = 1.00 m and (b) flow and three-dimensional thermal structures of original design and Temperature distribution at z = 1.00 m and d) flow and three-dimensional thermal structures of retrofitted design of AAU data center.

The retrofitting process was executed in a stepwise manner, with design modifications implemented sequentially to systematically evaluate their impacts on overall system performance. As illustrated in Fig. 11, one of the key modifications involved repositioning the server racks to achieve better alignment with the fan coil outlets. This adjustment was intended to establish a more direct and unobstructed cooling pathway, thereby reducing airflow resistance and ensuring that the cold air supplied by the fan coil units was efficiently utilized by the racks. To further optimize airflow distribution and containment, strategically placed airflow separators were introduced around the racks. These separators were designed to enforce a well-defined hot-aisle and cold-aisle containment strategy, effectively preventing undesired thermal mixing between the exhaust air from the servers and the cold supply air. By maintaining a clear separation between hot and cold zones, this configuration significantly improved cooling system efficiency, reduced the operational load on the fan coil units, and enhanced temperature uniformity across the data center. Additionally, further enhancements were implemented by incorporating additional airflow dividers to narrow the flow area and optimize airflow pathways. This modification minimized cold air bypass, ensuring that cooling was delivered precisely to the critical areas where it was needed most, thereby maximizing the overall effectiveness of the cooling strategy. Another critical design modification was sealing inlets of unused server bays to prevent leakage of cold air into inactive areas. This measure ensured that the cold supply air was directed exclusively through active servers before reaching the fan coil inlets. By eliminating unnecessary cooling losses and concentrating airflow on heat-generating components as illustrated Fig. 12, this modification further optimized cooling efficiency and contributed to a reduction in overall energy consumption. These studies were successively conducted by following the proposed KPI-informed enhancement process depicted in Fig. 6.

The results demonstrate that the proposed retrofitted design achieved a 75% improvement in the RTI and a 18% enhancement in the RHI (Table 8). Overall, these design modifications substantially improved thermal management by minimizing cooling inefficiencies and reducing temperature fluctuations. The refined airflow strategy resulted in a more stable and efficient cooling system, improving energy efficiency while mitigating the risk of localized hotspots. These findings highlight the critical role of systematic airflow optimization in developing sustainable and high-performance data center cooling solutions. Here, the RHI, RTI, and RI are indicators related to the direction and separation of airflow within the data center. These KPIs improve when cold and hot air streams remain isolated and when hot exhaust air is effectively directed toward the cooling units. However, RCI_HI_ may still decrease even under well-organized airflow conditions if the cooling capacity is insufficient or if the air delivered to the servers is already at lower than recommended temperatures. In such cases, although airflow management is efficient, the thermal conditions at the server inlets may still be below the recommended thresholds, leading to a lower RCI_HI_ value. In such cases, operational parameters of the cooling system such as supply flow rate and temperature should be optimized to maximize the RCI_HI_ value.

**Table 8:** KPI values for alternative designs after the application of retrofitted design.

KPI	Original	Retrofitted	Improvement (%)
RCI_HI_	205.80	215.80	−4.85
RCI_LO_	174.20	164.20	5.74
RTI	46.11	80.55	74.69
RHI	0.78	0.92	17.94
RI	97.21	99.57	2.42

## Conclusions

This study conducted a comprehensive analysis of thermal and cooling efficiencies in two distinct European data center environments located at university and supercomputing center. A robust numerical model was developed and validated to accurately represent the complex thermal dynamics and airflow patterns within these real-world settings by employing a combination of detailed experimental campaigns and advanced Computational Fluid Dynamics (CFD) simulations. The integration of key performance indicators (KPIs) provided a quantitative framework for assessing and comparing the thermal performance and cooling efficiency of both data centers, offering valuable insights into operational optimization and design enhancement.

Initial assessments revealed significant disparities in performance between PSNC and AAU data centers. The PSNC data center exhibited substantial deviations in KPIs from optimal ranges, largely attributed to suboptimal positioning of ventilation units and open-aisle hot and cold air mixing. In contrast, the AAU data center demonstrated relatively sound thermal performance, with KPIs generally falling within acceptable ranges. However, detailed analysis of the Return Temperature Index (RTI) and airflow analysis identified areas for potential improvement, particularly regarding hot air recirculation and cold air bypass around server racks. Based on these initial findings, targeted retrofitting strategies were developed and numerically evaluated for both data centers. The PSNC data center benefited significantly from the removal of ceiling-mounted ventilation units and the implementation of airflow management separators, effectively creating a hot and cold aisle containment system. This led to a 50% improvement in the Return Heat Index (RHI) and a 31% increase in the Recirculation Index (RI), highlighting the effectiveness of strategic geometric modifications. For the AAU data center, retrofitting involved repositioning server racks, adding airflow separators, and sealing unused server bays. These changes resulted in a 75% improvement in the RTI and a 18% increase in the RHI, demonstrating the considerable impact of refined airflow management strategies.

The outcomes of this study emphasize the critical importance of the CFD modeling and KPI-informed assessments in identifying and mitigating thermal inefficiencies in data centers. The implemented retrofitting approaches, validated through numerical simulations, led to significant enhancements in energy efficiency and thermal management within both pilot facilities. These findings provide a valuable framework for optimizing the performance of existing data centers, contributing to the broader goals of reducing energy consumption, minimizing carbon emissions, and aligning with the European Energy Efficiency Directive (EED). The methodologies and insights gained from this study are readily adaptable and can be applied to evaluate and improve the efficiency of a wide range of data center environments.

## Data Availability

All data generated or analysed during this study are included in this published article or available from the corresponding author on reasonable request.

## List of symbols

*D*: Darcy Coefficient
*F*: Forchheimer Coefficient
$$\:{g}_{i}$$: Gravitational Acceleration (m/s^2^)
*h*: Favre-averaged Enthalpy (kgm^2^/s^2^)
*k*: Turbulent Kinetic Energy (m^2^/s^2^)
$\:\mu\:\:\mu_t$: Molecular and Turbulent Viscosity (m^2^/s)
$\:\nu\:\:\nu_t$: Kinematic Viscosity, Turbulent Viscosity (m^2^/s)
*p*: Pressure (Pa)
*ρ*: Density (kg/m^3^)
$$\:{S}_{i}$$: Momentum Source
*t*: Time (s)
$\:{\varDelta\:T}_{equip}$: Temperature Difference of IT Equipments (°C)
$\:{T}_{in}$: Inlet Temperature of Server (°C)
$\:{T}^{max-rec}$: Maximum Recommended Temperature (°C)
$\:{T}^{max-all}$: Maximum Allowable Temperature (°C)
$\:{T}^{min-rec}$: Minimum Recommended Temperature (°C)
$\:{T}^{min-rec}$: Minimum Allowable Temperature (°C)
$$\:{T}_{sup}$$: Supply Temperature (°C)
$\:{T}_{rackInlet,mean}$: Mean Temperature of the Rack Inlets (°C)
*u*: Velocity (m/s)
$$\:{x}_{i}\:{x}_{j}$$: Cartesian Coordinates
*w*: Turbulence Viscosity (m^2^/s).

## Abbreviations

AAU: Aalborg University
ASHRAE: American Society of Heating, Refrigerating and Air-Conditioning Engineers
CFD: Computational Fluid Dynamics
EED: Energy Efficiency Directive
EUDCA: European Data Center Association
HEATWISE: Holistic Energy Management And Thermal Waste Integrated System For Energy Optimisation
HVAC: Heating, Ventilation and Air Conditioning
IT: Information Technology
PDU: Power Distribution Units
IPMI: Intelligent Platform Management Interface
KPI: Key Performance Indicator
PSNC: Poznan Supercomputing and Network Center
RCIHI: Rack Cooling Index, High
RCI_LO_: Rack Cooling Index, Low
RHI: Return Heat Index
RI: Recirculation Index
RTI: Return Temperature Index
SST: Shear Stress Transport
